# High variability in resistance to valgus deformation among knee braces indicated for medial collateral ligament injuries: a standardized bench-model biomechanical comparison

**DOI:** 10.1007/s00402-026-06519-6

**Published:** 2026-09-27

**Authors:** Evmorfia Pechlivanidou, Monika Stacheter, Peter Wahl, Matthias Jacobi

**Affiliations:** 1 Department of Hygiene, Epidemiology and Medical Statistics, Medical School, National and Kapodistrian University of Athens, Athens, Greece; 2 ARTORG Center for Biomedical Engineering Research, Faculty of Medicine, University of Bern, Bern, Switzerland; 3 Orthopädie Rosenberg, St Gallen, Switzerland; 4 Department of Biomedical Engineering, University of Basel, Allschwil, Switzerland; 5 Endo-Team, Birshof Hospital, Muenchenstein, Switzerland

**Keywords:** Orthoses, Joint laxity, Mechanical stiffness, Medical device regulation

## Abstract

**Introduction:**

To quantify valgus deformation and per-load mechanical stiffness of commercially available knee braces indicated for medial collateral ligament (MCL) injuries, and to evaluate whether mechanical performance is associated with material classification and qualitative stability assessment.

**Materials and methods:**

Nine knee braces identified through a national market survey were tested on a custom lower limb mechanical surrogate under progressive valgus loading at 5.7, 11.4, and 19.4 Nm. Angular deformation was measured using video-based motion tracking, and per-load brace stiffness (Nm/°) was computed at each load level.

**Results:**

At maximum applied torque (19.4 Nm), per-load stiffness ranged from 1.6 to 6.5 Nm/° across the nine devices — a fourfold difference between the stiffest and most compliant brace — with median valgus deformation of 8° (range 3–12°). In an exploratory, underpowered between-category comparison, rigid braces showed numerically higher stiffness than semi-rigid and soft devices (ε² = 0.34–0.45); this comparison is reported for hypothesis generation only. Qualitative ratings showed only moderate, inconsistent correlations with measured performance.

**Conclusions:**

Commercially available knee braces indicated for MCL injuries demonstrate highly variable mechanical resistance to valgus deformation under standardized bench conditions. The present sample was insufficient to determine whether material category accounts for this variability. These findings describe device-specific mechanical performance and do not constitute evidence for or against the clinical effectiveness of MCL bracing; they underscore the need for standardized biomechanical testing.

**Supplementary Information:**

The online version contains supplementary material available at https://doi.org/10.1007/s00402-026-06519-6.

## Introduction

 The medial collateral ligament (MCL) is the primary static restraint against valgus stress at the knee, comprising three distinct components: the superficial MCL, the deep MCL, and the posterior oblique ligament [1–4]. MCL injuries are among the most common ligamentous knee injuries in physically active individuals, with reported incidence rates of 0.24–7.3 per 1,000 person-years [5–8], occurring predominantly through valgus loading or pivoting mechanisms in contact sports, skiing, and tennis [5, 7, 9].

Contemporary understanding frames the medial side of the knee as a ligament complex rather than a single structure. The superficial MCL is the primary restraint to valgus load, whereas the deep MCL — also termed the anteromedial ligament — contributes predominantly to restraint of external rotation, and the posterior oblique ligament becomes increasingly important for posteromedial stability in extension [2, 10, 11]. Dynamic stabilisation is provided by the semimembranosus and its capsular expansions. Because these structures act synergistically, medial instability extends beyond isolated coronal valgus laxity to encompass anteromedial and posteromedial rotational components, and residual medial insufficiency increases strain on the anterior cruciate ligament and on ACL grafts [2, 12]. Current diagnostic and therapeutic algorithms therefore increasingly address rotational instability patterns rather than isolated valgus laxity. The present study characterises resistance to a single mechanical constraint within this complex — static valgus restraint, provided principally by the superficial MCL — and does not address the rotational or dynamic contributions of the remaining structures.

Non-operative management is the standard approach for most MCL injuries given the ligament’s inherent healing capacity [3, 9, 13]. Injuries are graded from I to III according to the Hughston classification [3, 14], with Grade II and III injuries commonly treated with hinged knee bracing [9, 13, 15–17]. Surgical intervention is reserved for displaced Grade III injuries, combined ligamentous injury, or persistent instability [12, 18]. Despite widespread clinical use, biomechanical studies have consistently demonstrated high variability in brace performance. Carlson and French reported an eightfold variation in stiffness across 11 braces (0.9–7.2 Nm/°) [19], while earlier cadaveric and benchtop work established that knee braces failed to consistently protect the MCL under valgus loading [20–22]. More recent comparative testing has confirmed marked between-device variability [23, 24]. Across nearly four decades of literature, no consensus mechanical-performance standard has emerged; brace effectiveness for MCL injuries has been rated as “very low” quality evidence [25], and the absence of standardised preclinical testing methods has been identified as a major regulatory gap in the peer-reviewed orthopaedic-device literature [26], within coverage and clearance frameworks that do not mandate such testing [15, 27].

The primary aim of this study was to quantify valgus angular deformation and per-load brace stiffness in commercially available knee braces indicated for MCL injury management under a standardised loading protocol. Secondary, exploratory aims were to assess the association between mechanical performance and qualitative clinical assessment, material classification, and load-dependent behaviour; given the limited number of eligible devices, these secondary analyses are hypothesis-generating rather than confirmatory.

## Materials and methods

### Study design and experimental setup

**Fig. 1:**
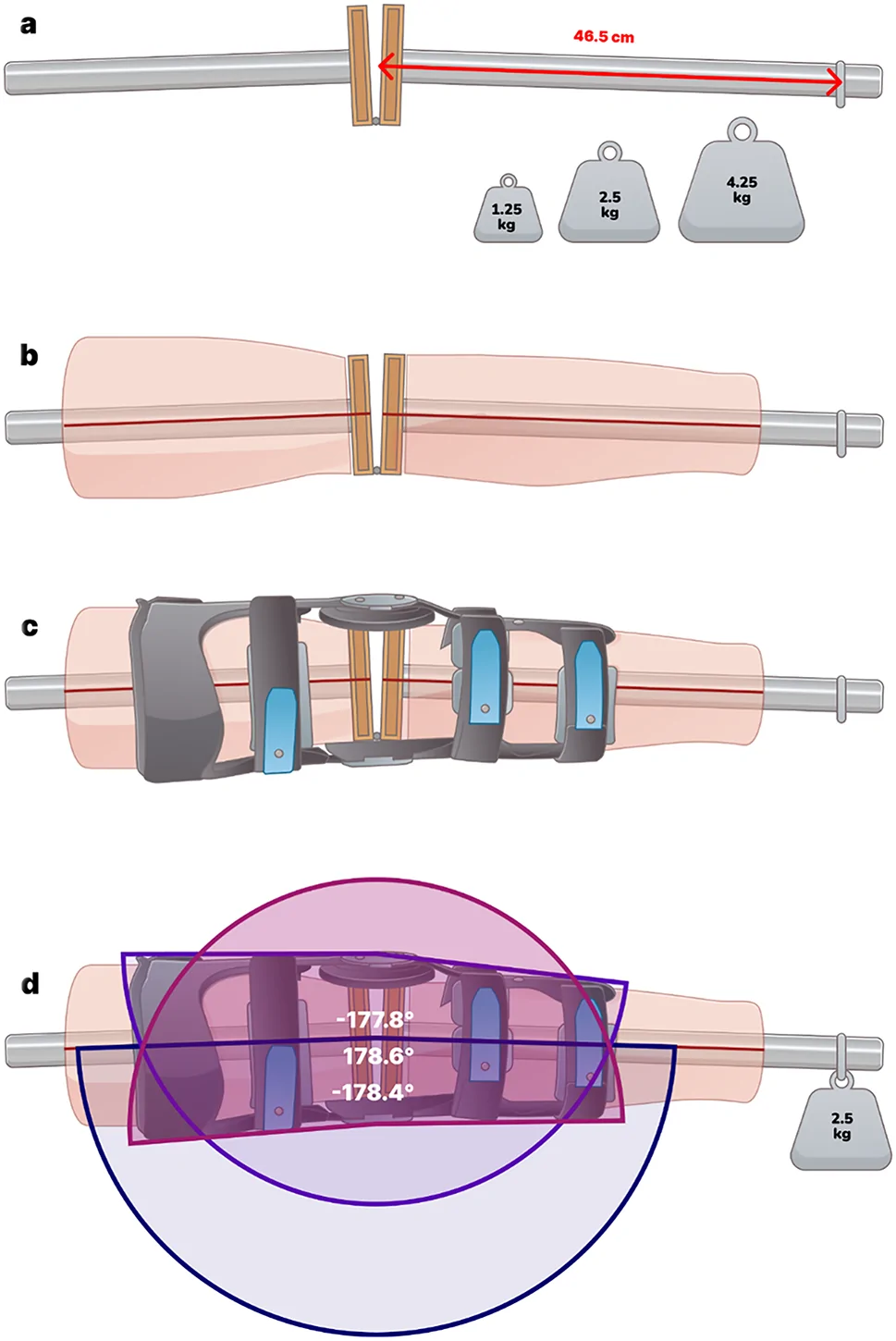
Schematic of the custom lower-limb model and experimental setup. **a** Aluminium tube framework with wooden knee-joint blocks, illustrating the 46.5 cm lever arm from the joint axis to the point of load application and the open medial compartment representing the simulated complete MCL deficiency; the three standardized masses (1.25, 2.5 and 4.25 kg) applied to generate the valgus torques are shown. **b** Lower-limb model with the polyethylene foam sleeve mounted on the framework. **c** Knee brace mounted on the leg model. **d** Magnified view during loaded testing (2.5 kg) showing the coordinate-based angular measurement obtained in Kinovea; valgus deformation is quantified as the change in the tibiofemoral angle relative to the unloaded baseline. A photograph of the assembled setup, with and without the superimposed measurement, is provided in Supplementary Fig. S1

A custom lower limb model was constructed to evaluate brace resistance to valgus deformation in isolation from other knee stabilisers, using the right leg of the second author (M.S.) as a geometric template. A single, fixed geometry was adopted deliberately: because every device was tested on identical dimensions, differences in measured deformation are attributable to the brace rather than to inter-limb variation, which would otherwise confound the comparison; the segment dimensions used fall within the range of published adult anthropometric data. The model deliberately omitted cruciate ligaments, menisci, capsular structures, and dynamic muscular control to isolate the brace’s mechanical contribution. Closed-cell polyethylene foam (Plastazote^®^, Zotefoams, Croydon, UK) simulated soft tissue elasticity. The model had a total length of 101.5 cm, with a tibial lever arm of 46.5 cm from the hinge centre to the point of load application (Fig. 1a).

The knee joint was replaced by a single-degree-of-freedom hinge with an open medial compartment, permitting rotation only in the coronal plane, to simulate complete MCL rupture. The femoral segment was mounted on an aluminium cylinder for mechanical stability. Unbraced and braced configurations are shown in Fig. 1b and c.

Deformation under load was recorded with a stationary 4 K camcorder (Panasonic HC-VX3; Panasonic, Osaka, Japan) recording at 30 frames per second, aligned orthogonally to the coronal plane of the model; orthogonality and inter-landmark distances were verified using a laser measure (Bosch Professional GLM 40-31; Bosch, Germany); the assembled configuration is shown photographically, with and without the superimposed angular measurement, in Supplementary Fig. S1. Brace position on the model was monitored throughout loading, and no slippage or migration of any device was observed. Controlled valgus torques of 5.7, 11.4, and 19.4 Nm were applied via suspended masses of 1.25, 2.5, and 4.25 kg at the defined lever arm, corresponding to clinically relevant loading conditions [28, 29]. These torques (calculated as τ = F × d) were chosen to span and slightly exceed the range over which comparable orthosis testing has historically been performed, permitting direct comparison with previously reported stiffness values [19], and lie well below the failure thresholds of the intact medial complex; the protocol therefore characterises brace behaviour in the sub-injury range.

Valgus deformation was defined as the change in tibiofemoral angle relative to the unloaded baseline, measured from three digitised landmark angles per test (Fig. 1d) using Kinovea 0.9.5 (open source). Each landmark angle was defined by three tracked points with the vertex at the middle point and computed from the tracked segment vectors as the difference of their orientations (arctan2 formulation) with wrap-around correction. The three angles corresponded to the central mechanical axis, the proximal/medial component, and the distal/lateral component. For each loading condition, the angle was read at the corresponding key-frame relative to the unloaded baseline and averaged across three repeated trials; between trials the brace was removed and re-donned and strap tension re-applied by the same operator, so that the repeated measurements capture re-application variability. Key-frame readings were verified against the source video and corrected where an isolated single-frame extraction error was identified. For each brace, the maximum deformation across the three angles was retained; the reported value for each brace and load level is therefore the mean across the three repeated trials at the landmark angle yielding maximum deformation. Because valgus deformation cannot decrease as applied torque increases, reported values were additionally constrained to be non-decreasing across the three load levels; this constraint was binding in two of 27 cells (SofTec Genu and MOS Genu at 19.4 Nm), in which the mean at maximum applied torque did not exceed the value recorded at the intermediate torque. Final values were rounded to the nearest integer degree, as sub-degree precision exceeds both the resolution of video-based measurement and clinical detectability.

### Brace selection and characteristics

A total of 20 braces from 10 manufacturers were identified through a national market survey in collaboration with a local orthotics team. Devices were selected for biomechanical testing to be size-compatible with the standardised model, to cover all three material categories, to be available as new, unused specimens, and to reflect the braces most frequently prescribed for MCL injuries in the collaborating regional practice (Table 1). Nine devices met these criteria and were analysed. Tested and non-tested devices did not differ significantly across the recorded characteristics (weight, length, price, and lockability; all *p* > 0.05), and manufacturer specifications for all 20 devices are provided in Supplementary Table S1. Braces were classified by application method (wrap-around or pull-on) and by frame material: rigid (aluminium, carbon-reinforced), semi-rigid (polymer, composite, hybrid), or soft (elastic, foam-textile, knit). Both classifications were determined prior to testing. Qualitative stiffness was rated independently by three of the authors (E.P., M.S., M.J.), blinded to the mechanical-test results and to one another’s ratings, using a five-point Likert-type scale [30] (1 = very unstable, 5 = very stable); majority rating was adopted, with consensus discussion where all three ratings differed.

**Table 1:** Overview of the selected knee braces and their structural characteristics

Brace name	Manufacturer	Clinical indication for MCL injuries^1^	Hinge Type^1^	Frame material^1^	Lockable^1^	Closure system^1^	Application method^2^	Material^3^	Testing status^4^
ADVAGO Knee	Oped	MCL/LCL/ACL injuries	Dual hinge	Composite with carbon elements	Yes	4 Velcro straps	Wrap-around	Rigid	Tested
Orthoteam Bodyfit Custom	Ortho Team	MCL grade I–II	Dual hinge	Synthetic mesh with metal bars	No	3–4 Velcro straps	Wrap-around	Semi-rigid	Tested
DonJoy Functional (Armor / Playmaker II)	DonJoy (DJO Global)	MCL/LCL/ACL injuries	Dual hinge	Aluminium/polymer hybrid	Yes	4 Velcro straps	Pull-on	Semi-rigid	Tested
Genu Arexa / 50K14	Ottobock	MCL/LCL injuries; postoperative rehabilitation	Dual hinge	Aluminium/polymer composite	Yes	4 Velcro straps	Wrap-around	Semi-rigid	Tested
Genudyn CI	Sporlastic	MCL/LCL grade II–III	Dual hinge	Composite frame	Yes	4 Velcro straps	Pull-on	Semi-rigid	Tested
Genudyn CI Novel	Sporlastic	MCL/LCL grade II–III	Dual hinge	Composite/carbon frame	Yes	4 Velcro straps	Wrap-around	Semi-rigid	Not tested
Genudyn CI Step Thru	Sporlastic	MCL grade I–II	Dual pivot hinge	Polymer frame	No	Slip-on + straps	Wrap-around	Semi-rigid	Not tested
Genutrain OA	Bauerfeind	MCL grade I	No hinge	Elastic knit with side bars	No	Slip-on + straps	Pull-on	Soft	Not tested
Go Up Open	Orthoservice	MCL grade II–III; postoperative rehabilitation	Dual hinge	Composite frame	Yes	4 Velcro straps	Wrap-around	Semi-rigid	Not tested
Go Up Pro	Orthoservice	MCL instability	Dual hinge	Carbon composite frame	Yes	4 Velcro straps	Wrap-around	Rigid	Not tested
M.4s Comfort	Medi	MCL/LCL grade II–III; postoperative rehabilitation	Dual hinge	Aluminium frame	Yes	4 Velcro straps	Pull-on	Semi-rigid	Not tested
MOS Genu	Bauerfeind	MCL grade III; combined injuries	Dual hinge	Aluminium with foam padding	Yes	4 Velcro straps	Wrap-around	Rigid	Tested
Optivo Knee	Oped	MCL grade II–III	Dual hinge	Polymer frame	Yes	4 Velcro straps	Pull-on	Semi-rigid	Not tested
Pavis Knee Brace	Pavis	MCL grade II; postoperative rehabilitation	Dual hinge	Textile/foam	Yes	4 Velcro straps	Pull-on	Soft	Tested
Rebound Dual	Össur	MCL/ACL combined injuries	Dual hinge	Aluminium/carbon composite	Yes	4 Velcro straps	Wrap-around	Rigid	Not tested
Rebound Dual Basic	Össur	MCL/LCL grade II	Dual hinge	Aluminium/polymer frame	Yes	4 Velcro straps	Pull-on	Semi-rigid	Not tested
Rebound Dual Custom	Össur	MCL/ACL/PCL injuries	Dual hinge	Carbon fiber frame	Yes	Custom straps	Wrap-around	Rigid	Not tested
SecuTec Genu	Bauerfeind	MCL grade III; combined ligament injuries	Dual hinge	Aluminium frame	Yes	4 Velcro straps	Wrap-around	Rigid	Not tested
SecuTec Genu Flex	Bauerfeind	MCL/LCL grade III; combined injuries	Dual hinge	Polymer frame	Yes	4 Velcro straps	Pull-on	Semi-rigid	Tested
SofTec Genu	Bauerfeind	MCL grade I–II	Dual hinge	Elastic knit with metal bars	No	Slip-on	Pull-on	Soft	Tested

^1^Per manufacturer specifications

^2^Wrap-around: longitudinal opening with strap closure; Pull-on: continuous sleeve

^3^A priori classification based on primary load-bearing structure: rigid (aluminium,* carbon-reinforced)*,* semi-rigid (polymer*,* composite*, hybrid), soft (elastic, foam-textile, knit)

^4^Tested: included in biomechanical testing; Not tested: market survey only

### Brace stiffness and load-response characteristics

Per-load brace stiffness (k, Nm/°) was calculated as applied torque divided by measured angular deformation at each load level, consistent with established reporting conventions [19]. Stiffness at maximum torque (19.4 Nm) was categorised descriptively as low (k < 2 Nm/°), moderate (2 ≤ k < 4), or high (k ≥ 4). Load-dependent behaviour was assessed using the Progressive Deformation Ratio (PDR), defined as deformation at 19.4 Nm divided by deformation at 5.7 Nm. The ratio of the corresponding torques (3.4) served as a descriptive threshold: PDR < 3.4 indicates progressive stiffening; PDR ≥ 3.4 indicates progressive compliance.

### Model scope and validation status

The surrogate was not validated against cadaveric specimens, was not calibrated against stress-radiographic measurements of physiological valgus laxity [31, 32], and was not intended to reproduce the behaviour of an MCL-deficient human knee. Its purpose is comparative rather than representational: identical geometry, applied torques, and imaging plane across all devices ensure that differences in measured deformation reflect the devices themselves. The model therefore supports rank-ordering and quantification of brace-specific static valgus restraint under standardized conditions, but does not support inference regarding in-vivo laxity, the behaviour of the medial ligament complex as a whole, or clinical efficacy. Testing was performed in full extension — the position in which these braces are applied during early protected mobilisation and in which their frames are designed to resist valgus load. Because the surrogate permits coronal-plane motion only, introducing flexion without rotation would correspond to no clinical examination position and was therefore not undertaken. Clinical assessment of the superficial and deep MCL is conventionally performed at 20–30° of flexion to isolate their contributions [2]; the present setup does not correspond to that position, and flexion-dependent brace behaviour was neither assessed nor can be inferred from these data.

### Statistical analysis

Descriptive statistics were reported as median, IQR, and range for continuous variables, and as counts and proportions for categorical data. No a priori sample-size calculation was performed; the sample was determined by the number of eligible devices available on the national market rather than by a power computation. Although inferential group comparisons were performed, the limited number of available devices rendered these analyses underpowered. They should therefore be interpreted as exploratory and hypothesis-generating. Non-parametric Wilcoxon rank-sum tests and Kruskal–Wallis tests were used for group comparisons of continuous variables and Fisher’s exact test for dichotomous variables, and correlations between continuous variables were assessed using Spearman’s rank correlation coefficient. A sensitivity analysis indicated that, at the realised category sizes, the achieved power of the three-group comparison was below 10%, and that approximately eight devices per category (≈ 24 in total) would be required for 80% power at α = 0.05; a true difference of the magnitude implied by the observed effect sizes would therefore be unlikely to reach significance in this sample. These comparisons are consequently interpreted as preliminary evidence requiring validation in a larger sample, and non-significant results are not read as evidence of absence of an effect. Effect sizes for group comparisons are reported as epsilon-squared (ε²) to inform sample-size planning for future work; at *n* = 9 they carry wide uncertainty and are not interpreted as evidence of a true underlying effect. Within-brace measurement reproducibility was characterized across the three repeated re-donned trials per load level, computed at the landmark angle yielding maximum deformation. All tests were two-sided; *p* < 0.05 was considered statistically significant. Statistical analyses were performed using R version 4.4.0 (R Foundation for Statistical Computing, Vienna, Austria).

## Results

### Valgus angular deformation under progressive load

Across all braces, valgus angular deformation increased progressively with applied torque. At the lowest level (5.7 Nm), median deformation was 3° (IQR 2–4°; range 1–5°); at 11.4 Nm, 7° (IQR 4–7°; range 3–8°); and at the maximum applied torque (19.4 Nm), median deformation reached 8° (IQR 5–10°; range 3–12°), demonstrating substantial inter-device variability. A fourfold difference in deformation was observed between the best- and worst-performing braces at maximum load.

**Fig. 2:**
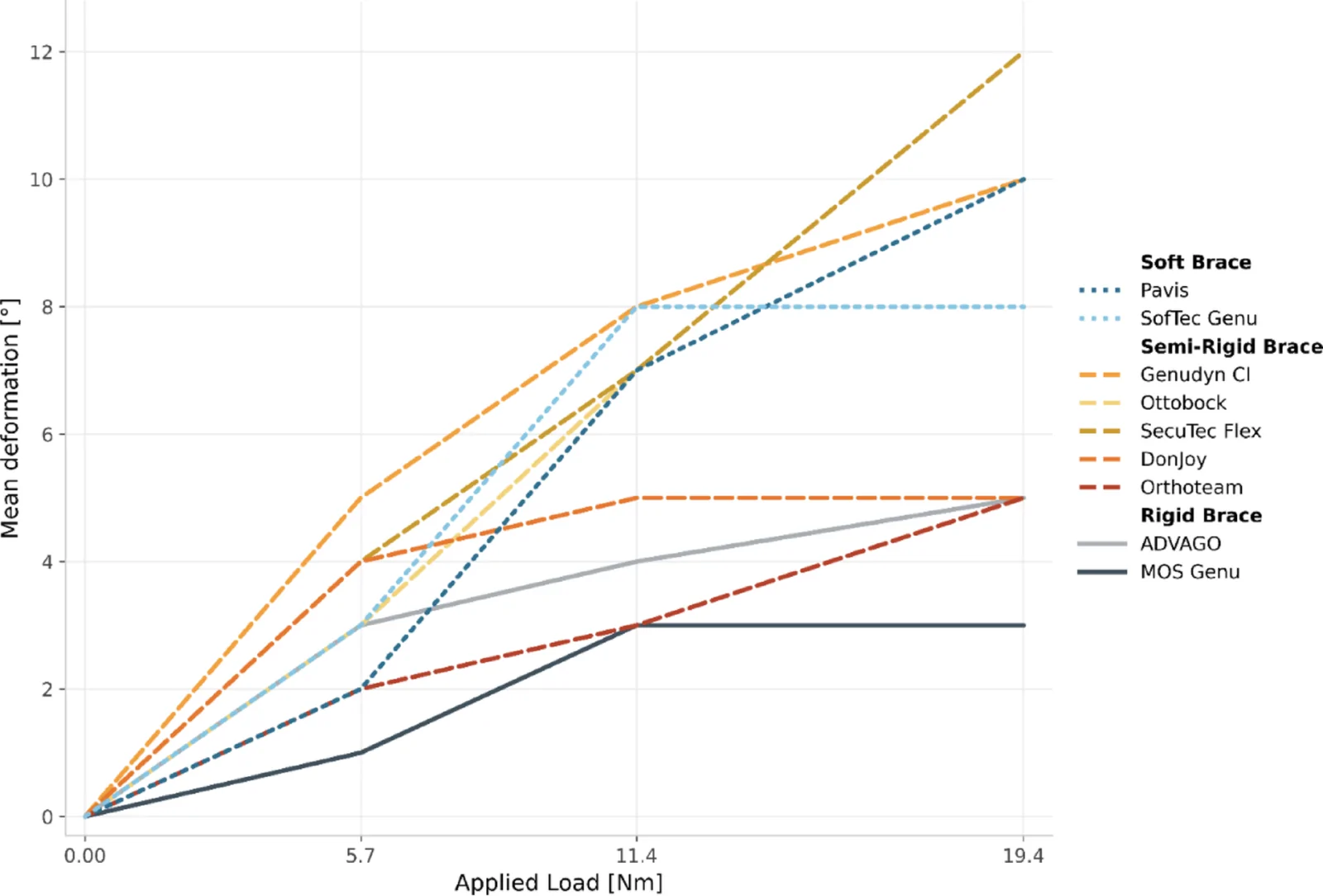
Valgus angular deformation across loading conditions for each tested knee brace. Bars represent the reported deformation value at each load level (5.7 Nm, 11.4 Nm, 19.4 Nm) per brace, as given in Table 2. Bars are color-coded and line-coded by brace and material category (rigid, semi-rigid, soft)

Measurement reproducibility differed markedly between devices. Across repeated trials at the landmark angle yielding maximum deformation, the median within-brace standard deviation was below 1° (IQR 0.4–1.7°), and five of the nine devices remained below 1.5° at every load level. Three cells showed substantially wider trial-to-trial spread, as the per-cell summaries in Table 2 show directly: the Genudyn CI at 11.4 and 19.4 Nm and the Genu Arexa / 50K14 at 11.4 Nm each spanned more than 4° across the three trials. Because the brace was removed, re-donned and re-tensioned by the same operator between trials, and because no slippage was observed within any individual trial, this spread reflects sensitivity of the device to re-application rather than instrument error; it is therefore reported per cell rather than pooled, so that device-specific fitting sensitivity remains visible and the reported values for these devices can be interpreted with correspondingly greater uncertainty. Individual brace deformation values are summarized in Table 2 and illustrated in Fig. 2.

**Table 2:** Mechanical deformation, stability metrics and qualitative classifications for all 9 tested knee braces

Brace	Deformation (°) ^1^			Stiffness (Nm/°)^2^	PDR^3^	QC^4^	Material^5^
	5.7 Nm	11.4 Nm	19.4 Nm				
ADVAGO Knee	**3** *2.6 (2.6–3.1)*	**4** *3.9 (3.5–3.9)*	**5** *5.2 (4.9–5.2)*	**3.9** moderate	**2** stiffening	**3**	Rigid
Orthoteam Bodyfit Custom	**2** *2.0 (1.5–2.0)*	**3** *3.6 (3.2–3.6)*	**5** *5.4 (4.3–5.4)*	**3.9** moderate	**2** stiffening	**4** (3–4)	Semi-rigid
DonJoy Functional	**4** *4.1 (3.7–4.4)*	**5** *4.3 (4.3–5.0)*	**5** *5.0 (3.7–5.9)*	**3.9** moderate	**1** stiffening	**4** (3–4)	Semi-rigid
Genu Arexa / 50K14	**3** *3.2 (2.8–3.3)*	**7** *8.2 (5.2–9.6)*	**12** *12.1 (12.1–12.2)*	**1.6** low	**4** compliance	**4** (4–5)	Semi-rigid
Genudyn^®^ CI	**5** *5.2 (5.0–5.5)*	**8** *10.3(5.6–11.5)*	**10** *8.8 (6.5–12.5)*	**1.9** low	**2** stiffening	**3**	Semi-rigid
MOS Genu	**1** *0.9 (0.9–1.1)*	**3** *2.3 (2.3–3.0)*	**3** *2.1 (1.2–2.1)*	**6.5** high	**3** stiffening	**5**	Rigid
Pavis Knee Brace	**2** *3.2 (1.9–3.2)*	**7** *7.2 (6.3–8.4)*	**10** *8.9 (8.1–11.3)*	**1.9** low	**5** compliance	**2** (1–2)	Soft
SofTec^®^ Genu	**3** *3.2 (3.2–3.3)*	**8** *7.6 (7.6–7.7)*	**8** *4.7 (4.7–5.3)*	**2.4** moderate	**3** stiffening	**1**	Soft
SecuTec^®^ Genu Flex	**4** *4.2 (3.7–4.6)*	**7** *6.8 (6.5–7.0)*	**12** *12.0 (11.6–12.2)*	**1.6** low	**3** stiffening	**2**	Semi-rigid
Overall Median (IQR; range)¹·⁶	**3** (2–4; 1–5)	**7** (4–7; 3–8)	**8** (5–10; 3–12)	**2.4** (1.9–3.9; 1.6–6.5)	**3** (2–3; 1–5)	**3** (2–4; 1–5)	—

— not applicable

¹Valgus angular deformation (°). Reported value (bold) is the mean across the three repeated trials at the landmark angle yielding maximum deformation of the three measured angles, rounded to the nearest degree and constrained to be non-decreasing with applied torque (see Methods). Italic value below is the median (IQR₂₅–IQR₇₅) of the same three trials. Because the bold value is a mean and the italic summary a median, the two may differ; for SofTec Genu and MOS Genu at 19.4 Nm the bold value additionally reflects the non-decreasing constraint and therefore exceeds the corresponding trial median

² Per-load brace stiffness, k = applied torque ÷ measured angular deformation, reported at the maximum applied torque (19.4 Nm); stiffness at lower torques is obtained as torque ÷ deformation from the columns above. Categories: low (k < 2 Nm/°), moderate (2 ≤ k < 4), high (k ≥ 4)

³Progressive Deformation Ratio (PDR): deformation at 19.4 Nm ÷ deformation at 5.7 Nm. Descriptive threshold = 3.4 (ratio of the corresponding applied torques); stiffening = PDR < 3.4 (resistance increases with load); compliance = PDR ≥ 3.4 (resistance decreases with load). Per-brace PDR values are shown rounded to the nearest integer and the summary row is computed from these rounded values; computed from unrounded deformation ratios the median is 2.7 (range 1.2–5.0), as reported in the Results

⁴Qualitative consensus stability score (1–5 Likert; 1 = very unstable, 5 = very stable); majority vote of three independent blinded raters. Range in parentheses indicates inter-rater disagreement

⁵Frame classification based on primary load-bearing structure per manufacturer specifications: rigid (aluminium, carbon-reinforced), semi-rigid (polymer, composite, hybrid), soft (elastic, foam-textile, knit)

⁶Overall median (IQR; range) across all nine braces

Rank ordering was broadly but not fully preserved across load levels. Two devices changed position substantially: the Pavis Knee Brace and the Genu Arexa / 50K14 were among the least deformable devices at 5.7 Nm (2° and 3°, respectively) yet among the most deformable at 19.4 Nm (10° and 12°), whereas the DonJoy Functional showed the opposite pattern.

In an exploratory between-category comparison, rigid braces showed numerically lower deformation than the other categories across all load levels: at maximum applied torque, rigid braces demonstrated the lowest median deformation (4°; individual values 3° and 5°; *n* = 2), followed by semi-rigid (10°; IQR 5–12°; range 5–12°; *n* = 5) and soft braces (9°; individual values 8° and 10°; *n* = 2). Differences between material categories did not reach statistical significance at any load level (Kruskal–Wallis *p* > 0.15), with large effect sizes (ε² = 0.34–0.45); given the achieved power below 10%, these effect sizes are reported as sample-size planning inputs and are not interpreted as evidence of a true category difference. Within-category variability was most pronounced among semi-rigid braces, whose individual deformation values spanned the full range of results.

Application method was compared descriptively between wrap-around (*n* = 4) and pull-on (*n* = 5) devices. Median deformation was lower among wrap-around braces at every load level, but the two subgroups overlapped across almost the entire range of measured values. At 5.7 Nm: median 2.5° (IQR 1.8–3.0°; range 1–3°) versus 4.0° (IQR 3.0–4.0°; range 2–5°); at 11.4 Nm: 3.5° (IQR 3.0–4.8°; range 3–7°) versus 7.0° (IQR 7.0–8.0°; range 5–8°); at 19.4 Nm: 5.0° (IQR 4.5–6.8°; range 3–12°) versus 10.0° (IQR 8.0–10.0°; range 5–12°). At maximum applied torque the wrap-around range therefore spanned the full range of results observed in the study, as the device with the largest deformation (Genu Arexa / 50K14, 12°) is a wrap-around design; conversely, the lowest deformation recorded among pull-on devices (5°) matched that of two wrap-around devices. Differences between application methods did not reach statistical significance at any load level (all *p* > 0.05). Application method alone therefore did not separate devices by mechanical performance in this sample.

### Brace stiffness and load-response characteristics

Per-load brace stiffness varied substantially across devices and load levels. At 5.7 Nm, median stiffness was 1.9 Nm/° (IQR 1.4–2.9; range 1.1–5.7); at 11.4 Nm, 1.6 Nm/° (IQR 1.6–2.9; range 1.4–3.8); and at the maximum applied torque (19.4 Nm), 2.4 Nm/° (IQR 1.9–3.9; range 1.6–6.5). The observed range at maximum applied torque is consistent with the 0.9–7.2 Nm/° range previously reported by Carlson and French across 11 designs [19]. Of the nine braces, one corresponded to high stiffness (k ≥ 4 Nm/° at 19.4 Nm), four to moderate, and four to low. Rigid braces demonstrated the highest stiffness at maximum applied torque (median 5.2 Nm/°; individual values 3.9 and 6.5; *n* = 2), followed by semi-rigid (median 1.9 Nm/°; range 1.6–3.9; *n* = 5) and soft braces (median 2.2 Nm/°; individual values 1.9 and 2.4; *n* = 2); these differences corresponded to large effect sizes (ε² = 0.34–0.45) but did not reach statistical significance (Kruskal–Wallis *p* > 0.15) and are, as above, interpreted as exploratory.

**Fig. 3:**
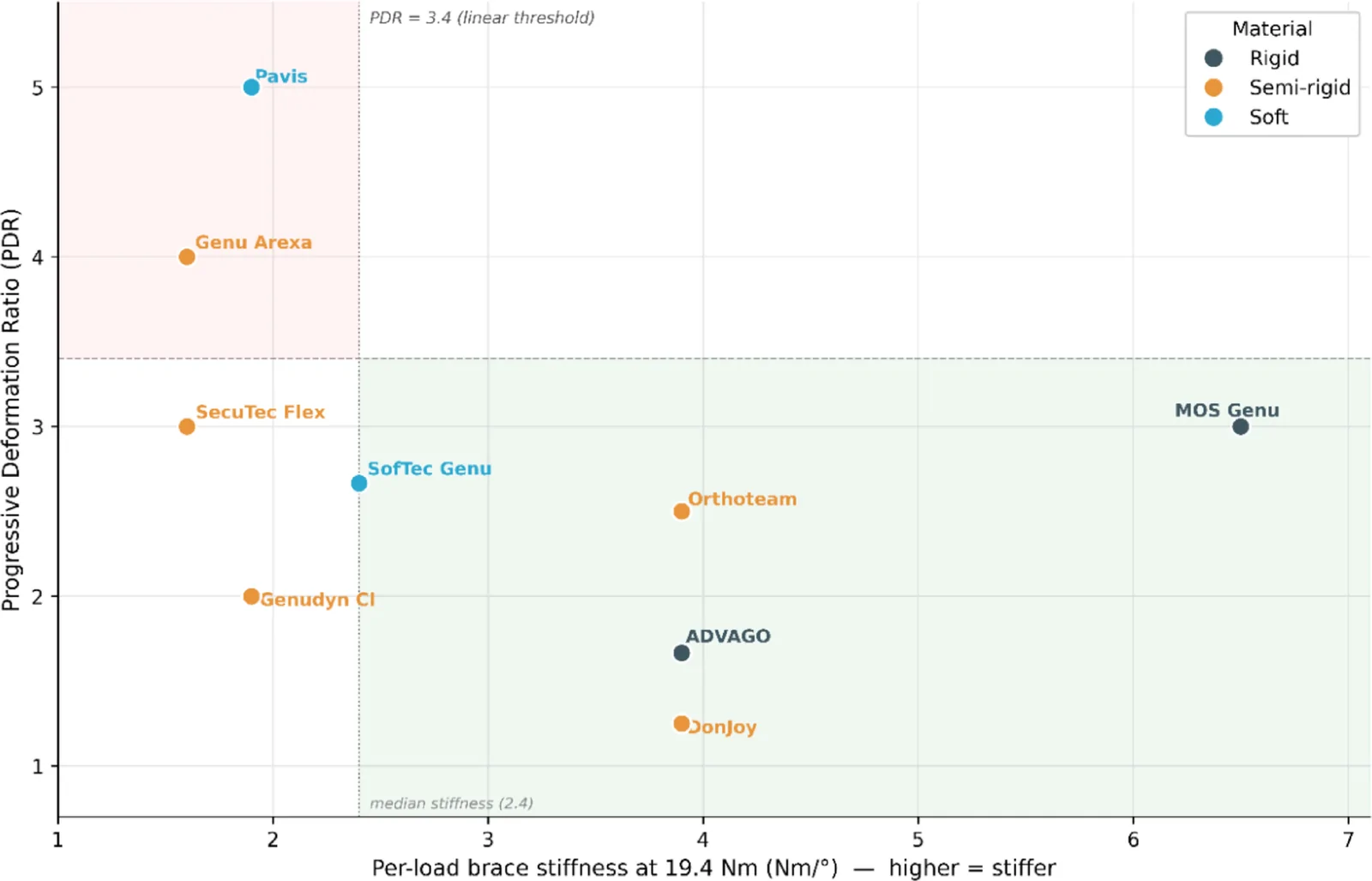
Mechanical behaviour classification based on per-load brace stiffness and load-response characteristics. Higher stiffness reflects lower valgus deformation under load. The horizontal dashed line represents the descriptive linear-behaviour threshold (PDR = 3.4). Braces above the line demonstrate progressive compliance; braces below demonstrate progressive stiffening

PDR ranged from 1.2 to 5.0 (median 2.7). Seven braces exhibited progressive stiffening behaviour (PDR < 3.4), whereas two demonstrated progressive compliance (PDR ≥ 3.4), the soft Pavis Knee Brace and the semi-rigid Genu Arexa / 50K14, both of which were among the devices with the largest deformation magnitudes at maximum applied torque. As no device slippage was observed during testing, this progressive compliance is attributable to intrinsic load-dependent behaviour — plausibly progressive give at the strap or hinge interface and compression of the compliant interface — rather than gross fitting failure; PDR is reported as a descriptive index only. Per-brace stiffness and PDR values are reported in Table 2 and illustrated in Fig. 3.

### Consistency of mechanical performance across load levels

Spearman correlation analysis demonstrated positive correlations between deformation values across load levels (ρ = 0.6 between 5.7 Nm and 11.4 Nm; ρ = 0.5 between 5.7 Nm and 19.4 Nm; ρ = 0.7 between 11.4 Nm and 19.4 Nm), indicating moderate to strong, though incomplete, consistency of rank ordering under progressive loading.

### Inter-rater reliability of qualitative stability assessment

Inter-rater agreement among the three independent assessors was high regarding the qualitative assessment of stability. Pairwise Spearman correlation coefficients ranged from ρ = 0.87 to ρ = 0.95, indicating excellent consistency. The aggregated consensus score was used for all subsequent analyses.

### Qualitative stability ratings

Qualitative stability ratings had a median score of 3 (IQR: 2–4; range: 1–5). Rigid braces received the highest ratings (median 4; individual values 3 and 5), followed by semi-rigid (median 4; IQR 3–4; range 2–4), while soft braces demonstrated lower perceived stability (median 1.5; individual values 1 and 2).

### Association between qualitative and quantitative measures

**Fig. 4:**
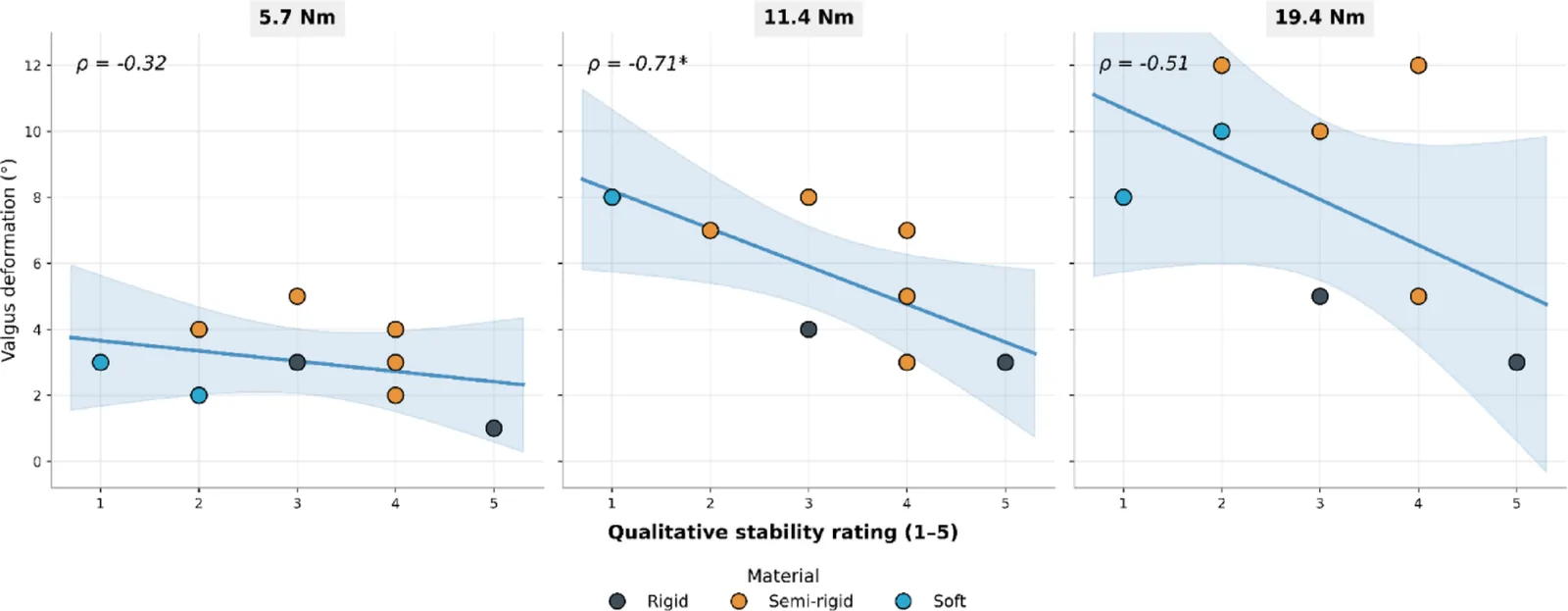
Association between qualitative stability ratings and measured valgus deformation across loading conditions. Qualitative stability was assessed independently by three blinded raters using a 5-point Likert-type scale (1 = very unstable, 5 = very stable). Spearman correlation coefficients: ρ = −0.3 at 5.7 Nm, ρ = −0.7 at 11.4 Nm (*p* = 0.03), ρ = −0.5 at 19.4 Nm. Shaded area represents 95% confidence interval

Qualitative stability scores were positively correlated with per-load brace stiffness across all load levels (Spearman ρ = 0.3 at 5.7 Nm, ρ = 0.7 at 11.4 Nm, ρ = 0.5 at 19.4 Nm), with corresponding negative correlations against measured deformation (ρ = −0.3, −0.7, −0.5). The association reached statistical significance only at 11.4 Nm (*p* = 0.03) and is illustrated in Fig. 4.

## Discussion

The present study provides a standardized biomechanical evaluation of commercially available knee braces indicated for the treatment of MCL injuries and demonstrates several clinically and conceptually relevant findings. Most notably, all tested braces exhibited measurable valgus deformation under applied torque, ranging from 3° to 12°, corresponding to per-load stiffness values from 1.6 to 6.5 Nm/° at maximum applied torque. Although substantial inter-device variability was observed, even the best-performing brace allowed a deformation of 3° at the maximum applied loading. Whether angular deformations of this magnitude are clinically consequential cannot be determined from a bench model and requires in-vivo investigation.

A key result of this study is the lack of concordance between qualitative clinical assessment and quantitative biomechanical performance. Qualitative stability ratings demonstrated only moderate and inconsistent correlations with measured stiffness and deformation (|ρ| = 0.3 to 0.7 across load levels), reaching statistical significance at only one of three load levels. Hands-on perception of rigidity likely reflects overall build quality and sagittal or torsional stiffness rather than coronal-plane valgus restraint specifically, which may account for this disconnect. This finding indicates that subjective assessment alone cannot reliably predict mechanical performance, supporting the need for objective biomechanical testing, as previously suggested in studies on knee stability assessment [23, 33, 34].

Rigid braces showed numerically lower deformation than the other categories, but this exploratory, underpowered comparison did not reach statistical significance and should not be read as establishing a material-class effect; the substantial within-category variability — particularly among semi-rigid braces, which spanned the full range of results — reinforces this caution. These findings suggest that material composition alone is insufficient to predict mechanical behaviour, and that design features, joint architecture, and load transfer mechanisms are likely more relevant determinants of brace performance, although a formal multivariable analysis of these design features as predictors was precluded by the present sample size and is identified as a priority for adequately powered future studies [10, 23].

An additional descriptive observation was that median valgus deformation was lower among wrap-around than among pull-on designs at every load level. This difference should not be over-interpreted: the two subgroups overlapped across almost the entire range of measured values, the device with the largest deformation at maximum applied torque was itself a wrap-around design, and the differences did not reach statistical significance. Whether application method influences load transfer — for example through circumferential tightening and more uniform soft-tissue capture, which might reduce device migration relative to pull-on sleeves [23, 24] — cannot be resolved by the present data and would require a purpose-designed comparison with adequate numbers of devices in each subgroup.

These findings should also be read against contemporary concepts of the medial ligament complex. Because the model isolates coronal-plane valgus restraint, it does not capture the contributions of the deep MCL and posterior oblique ligament to rotational stability, nor the dynamic role of the semimembranosus. A device that restrains coronal valgus may or may not restrain anteromedial or posteromedial rotational instability — a question the present setup cannot address. Frame material, moreover, is a coarse descriptor: hinge configuration, cuff length, strap geometry, and the resulting load-transfer path plausibly exert greater influence on valgus restraint than material class alone, an interpretation consistent with the failure of material category to account for the observed variability in this sample.

The clinical relevance of these findings must be interpreted in the context of physiological joint loading. The applied torques (5.7–19.4 Nm) were deliberately conservative; musculoskeletal modelling estimates the knee adduction moment during level walking at approximately 40–50 Nm [28, 29], and demands increase further during stair climbing, rehabilitation exercises, and sporting activities [28, 29]. These estimates arise from different biomechanical conditions and are not directly comparable to the externally applied torques used here; they are cited solely to indicate the order of magnitude of loading reported during physiological activity. By geometric conversion, at an average knee joint width of 60 mm, 1° of valgus deformation corresponds to approximately 1 mm of medial joint line opening [35]. Even the minimum observed deformation of 3° thus corresponds to approximately 3 mm of medial opening in the absence of ligamentous restraint — a magnitude consistent with cadaveric reports of complete MCL disruption on stress radiography [31, 32]. At the upper end, the 12° deformation of the worst-performing brace corresponds to approximately 12 mm of opening, consistent with severe ligamentous instability. Whether such mechanical laxity translates into adverse clinical outcomes warrants further prospective investigation [9, 11]. We do not propose a clinically acceptable threshold of valgus laxity; the geometric and cadaveric comparisons serve only to scale the measured deformations, and the present findings constitute a mechanical characterisation without clinical validation.

Objective, device-level mechanical data of this kind remain limited in the literature yet would be required inputs for any rigorous clinical comparison of bracing strategies. The present findings supply such data but do not themselves constitute clinical evidence: they neither support nor question the clinical effectiveness of MCL bracing, which remains supported only by low-quality evidence [16, 25, 36] and must be established through prospective clinical study.

Several limitations should be acknowledged. The simplified mechanical model deliberately omitted the cruciate ligaments, menisci, capsular structures, and active musculature to isolate the brace’s mechanical contribution, but this precludes direct extrapolation to the MCL-deficient knee in vivo. Because the model isolates the brace as the sole coronal-plane restraint, the protocol represents a worst-case condition that maximises the mechanical demand on the device; in the intact or partially intact knee, secondary restraints would share the valgus load and absolute deformation would be smaller. Findings should therefore be interpreted as characterising brace mechanical performance under standardised loading rather than knee-complex behaviour during functional activity. Testing was performed in knee extension, and brace behaviour may differ at higher flexion angles, where fixation transitions from a compression-based to a torsion-dominant mode, likely resulting in reduced frame stability. The surrogate represents a single anthropometry; brace fit, and therefore measured stiffness, may differ on limbs of different circumference or length, and the rank-ordering reported here should not be assumed invariant across body habitus. The model permits coronal-plane motion only, so anteromedial and posteromedial rotational instability were not assessed. The surrogate was not validated against cadaveric specimens or stress-radiographic data, and clinical efficacy cannot be inferred from these results. Cyclic loading, fatigue effects, and long-term performance were not assessed. Each device was tested in three repeated trials per load level; while this permits characterisation of re-application variability, it constrains the precision with which within-device variability can be estimated. The limited number of tested devices constrained statistical power for subgroup analyses and limits generalisability to other markets. Furthermore, fundamental clinical questions — including optimal indications for brace use, duration of bracing, permitted weight-bearing, and brace–skin interaction — remain insufficiently defined in the literature and were beyond the scope of this study [2, 9, 11, 13, 25].

In conclusion, under standardized bench conditions, commercially available knee braces indicated for MCL injury demonstrate highly variable resistance to static valgus deformation. Only static valgus restraint was investigated; rotational stability was not examined, and the present sample was insufficient to determine whether material category accounts for the observed variability. These results describe device-specific mechanical performance and should not be interpreted as evidence either supporting or questioning the clinical effectiveness of MCL bracing. Future research should prioritise standardised biomechanical testing, objective in-vivo stability assessment including rotational instability, and high-quality clinical studies to define the role of knee bracing in MCL injury management.

## Supplementary Information

Below is the link to the electronic supplementary material.

Supplementary Material 1. Supplementary Fig. S1 Experimental setup, loading configuration and angular measurement. (a) Photograph of the assembled surrogate with a representative brace in place, without measurement, showing the digitised tracking landmarks on the proximal and distal segments. (b) The same view at maximum applied torque with the Kinovea angular measurement superimposed; the on-screen angles are indicative single-trial readings, whereas the deformation values reported in the main text (Table 2) are the mean of three repeated trials. (c) Annotated schematic of the loading configuration: the applied force vector, the 46.5 cm lever arm from the joint axis to the point of load application, the resulting valgus torques (τ = F × d; 5.7, 11.4 and 19.4 Nm for applied masses of 1.25, 2.5 and 4.25 kg), and the open medial compartment representing the simulated complete MCL deficiency. (d) Definition of the angular measurement: α is the coronal-plane angle P1–P2–P3 between the femoral and tibial axes, and valgus deformation is the change from the unloaded baseline, Δα = α(baseline) − α(loaded)

Supplementary Material 2

## Data Availability

The datasets generated and analysed during the current study are available from the corresponding author on reasonable request.
