# Supplementary material for: High variability in resistance to valgus deformation among knee braces indicated for medial collateral ligament injuries: a standardized bench-model biomechanical comparison
Source: Arch Orthop Trauma Surg. 2026 Sep 27;146(1):348. doi: 10.1007/s00402-026-06519-6 (PMC13616848; doi:10.1007/s00402-026-06519-6)
Supplement: Supplementary file 1 [file 402_2026_6519_MOESM1_ESM.pdf]

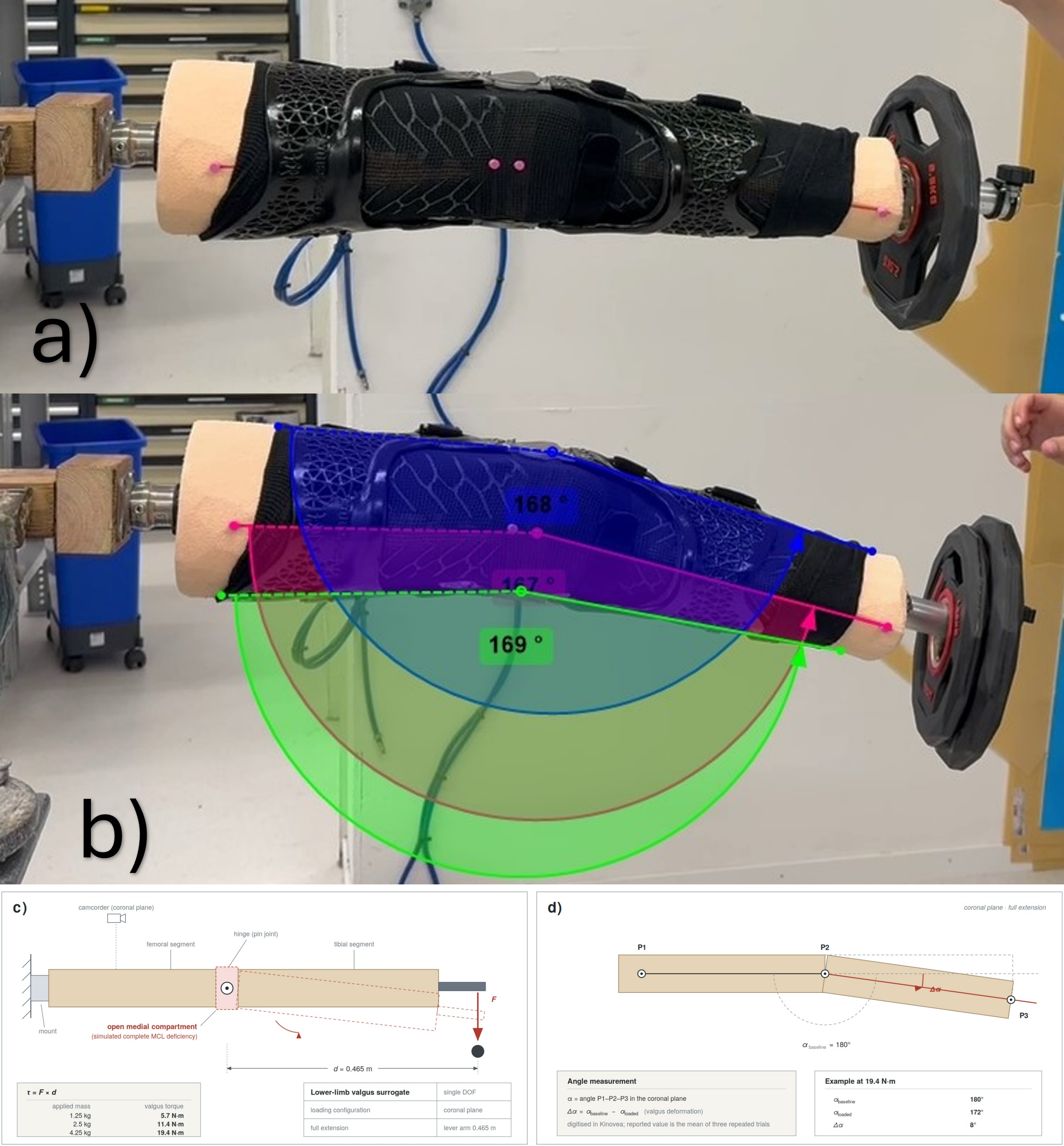

a)
b)
168 °
167 °
169 °
c)
camcorder (coronal plane)
femoral segment
hinge (pin joint)
tibial segment
F
mount
open medial compartment
(simulated complete MCL deficiency)
d = 0.465 m
τ = F × d
applied mass | valgus torque
1.25 kg | 5.7 N·m
2.5 kg | 11.4 N·m
4.25 kg | 19.4 N·m
Lower-limb valgus surrogate | single DOF
loading configuration | coronal plane
full extension | lever arm 0.465 m
d)
coronal plane · full extension
P1
P2
P3
Δα
α baseline = 180°
Angle measurement
α = angle P1–P2–P3 in the coronal plane
Δα = α_baseline − α_loaded (valgus deformation)
digitised in Kinovea; reported value is the mean of three repeated trials
Example at 19.4 N·m
α_baseline 180°
α_loaded 172°
Δα 8°
