# Supplementary material for: High variability in resistance to valgus deformation among knee braces indicated for medial collateral ligament injuries: a standardized bench-model biomechanical comparison
Source: Arch Orthop Trauma Surg. 2026 Sep 27;146(1):348. doi: 10.1007/s00402-026-06519-6 (PMC13616848; doi:10.1007/s00402-026-06519-6)
Supplement: Supplementary file 2 [file 402_2026_6519_MOESM2_ESM.docx]

**Supplementary Table S1.** Manufacturer specifications of all 20 knee braces identified in the market survey

| **Brace** | **Manufacturer** | **Frame material** | **Lockable** | **Weight (g)** | **Length (cm)** | **Price (CHF)** | **Application** | **Tested** |
| --- | --- | --- | --- | --- | --- | --- | --- | --- |
| ADVAGO Knee | Oped | Injection-moulded composite with carbon elements | Yes | 650 | 45 | 950 | Wrap-around | Yes |
| Orthoteam Bodyfit Custom | Ortho Team | Synthetic mesh with internal metal bars | No | 400 | 40 | 650 | Wrap-around | Yes |
| DonJoy Functional (Armor / Playmaker II) | DonJoy (DJO Global) | Aluminium/polymer hybrid frame with foam pads | Yes | 550 | 45 | 750 | Pull-on | Yes |
| Genu Arexa / 50K14 | Ottobock | Aluminium/polymer composite with foam padding | Yes | 650 | 45 | 800 | Wrap-around | Yes |
| Genudyn® CI | Sporlastic | Composite frame with foam interior | Yes | 500 | 45 | 780 | Pull-on | Yes |
| Genudyn® CI Novel | Sporlastic | Composite/carbon lightweight frame | Yes | 500 | 45 | 820 | Wrap-around | No |
| Genudyn® CI Step Thru | Sporlastic | Polymer frame with side bars | No | 450 | 40 | 680 | Wrap-around | No |
| Genutrain® OA | Bauerfeind | Elastic knit with lateral bars and unloading strap | No | 700 | 35 | 280 | Pull-on | No |
| Go Up Open | Orthoservice | Composite frame with anterior opening | Yes | 700 | 45 | 1350 | Wrap-around | No |
| Go Up Pro | Orthoservice | Carbon composite frame | Yes | 650 | 45 | 1500 | Wrap-around | No |
| M.4s® Comfort | Medi | Aluminium frame with AirFlow padding | Yes | 400 | 45 | 850 | Pull-on | No |
| MOS Genu | Bauerfeind | Aluminium with neoprene/foam padding | Yes | 650 | 45 | 900 | Wrap-around | Yes |
| Optivo Knee | Oped | Polymer frame with soft lining | Yes | 500 | 40 | 780 | Pull-on | No |
| Pavis Knee Brace | Pavis | Foam-textile with lateral hinge system | Yes | 650 | 40 | 450 | Pull-on | Yes |
| Rebound® Dual | Össur | Aluminium/carbon composite | Yes | 650 | 45 | 850 | Wrap-around | No |
| Rebound® Dual Basic | Össur | Aluminium/polymer frame with foam padding | Yes | 650 | 45 | 700 | Pull-on | No |
| Rebound® Dual Custom | Össur | Carbon fibre anatomical frame | Yes | 650 | 50 | 1100 | Wrap-around | No |
| SecuTec® Genu | Bauerfeind | Aluminium frame with neoprene/foam padding | Yes | 650 | 45 | 920 | Wrap-around | No |
| SecuTec® Genu Flex | Bauerfeind | Polymer frame with traction-pattern surface | Yes | 350 | 45 | 950 | Pull-on | Yes |
| SofTec® Genu | Bauerfeind | Elastic knit sleeve with metal side bars | No | 300 | 35 | 350 | Pull-on | Yes |

*Specifications per manufacturer documentation; devices are listed in the same order as Table 1. “Tested” indicates inclusion in the biomechanical testing (nine analysed devices); the remaining eleven devices were surveyed only. Tested and non-tested devices did not differ significantly in weight (p = 0.22), length (p = 0.62) or price (p = 0.34; Mann–Whitney U tests), or in lockability (p = 1.00; Fisher’s exact test).*
